# Structural characterization of the bacterial proteasome homolog BPH reveals a tetradecameric double-ring complex with unique inner cavity properties

**DOI:** 10.1074/jbc.M117.815258

**Published:** 2017-11-28

**Authors:** Adrian C. D. Fuchs, Lorena Maldoner, Katharina Hipp, Marcus D. Hartmann, Jörg Martin

**Affiliations:** From the ‡ Department of Protein Evolution and; § Electron Microscopy Facility, Max Planck Institute for Developmental Biology, Spemannstraße 35, 72076 Tübingen, Germany

**Keywords:** proteasome, protein crystallization, protein degradation, protein evolution, protein structure, small-angle X-ray scattering (SAXS), Anbu, BPH, HslV

## Abstract

Eukaryotic and archaeal proteasomes are paradigms for self-compartmentalizing proteases. To a large extent, their function requires interplay with hexameric ATPases associated with diverse cellular activities (AAA+) that act as substrate unfoldases. Bacteria have various types of self-compartmentalizing proteases; in addition to the proteasome itself, these include the proteasome homolog HslV, which functions together with the AAA+ HslU; the ClpP protease with its partner AAA+ ClpX; and Anbu, a recently characterized ancestral proteasome variant. Previous bioinformatic analysis has revealed a novel bacterial member of the proteasome family Betaproteobacteria proteasome homolog (BPH). Using cluster analysis, we here affirmed that BPH evolutionarily descends from HslV. Crystal structures of the *Thiobacillus denitrificans* and *Cupriavidus metallidurans* BPHs disclosed a homo-oligomeric double-ring architecture in which the active sites face the interior of the cylinder. Using small-angle X-ray scattering (SAXS) and electron microscopy averaging, we found that BPH forms tetradecamers in solution, unlike the dodecamers seen in HslV. Although the highly acidic inner surface of BPH was in striking contrast to the cavity characteristics of the proteasome and HslV, a classical proteasomal reaction mechanism could be inferred from the covalent binding of the proteasome-specific inhibitor epoxomicin to BPH. A ligand-bound structure implied that the elongated BPH inner pore loop may be involved in substrate recognition. The apparent lack of a partner unfoldase and other unique features, such as Ser replacing Thr as the catalytic residue in certain BPH subfamilies, suggest a proteolytic function for BPH distinct from those of known bacterial self-compartmentalizing proteases.

## Introduction

Self-compartmentalizing proteases are found in all kingdoms of life. Paradigm is the proteasome, a barrel-shaped complex of four stacked rings with 7-fold symmetry (1). Its activity resides in the β subunits that form the inner rings, whereas the outer rings consist of catalytically inactive α subunits. Both types of subunits are similar in sequence and structure and are thought to have evolved by duplication of an ancestral proto-β subunit (2). In eukaryotes, this so-called 20S proteasome is complemented by accessory interaction partners, in particular the 19S lid particle, which is responsible for substrate recognition, ATP-dependent unfolding, and translocation into the core of the proteasomal cylinder (3). Together, this assembly forms the 26S proteasome. Also, ATP-independent functions of the 20S form have been discussed to be relevant for the physiology of the eukaryotic proteasome (4–7).

Although the eukaryotic proteasome is a very complex representative of self-compartmentalizing proteases, its evolution started from more simpler versions, and, indeed, archaea have a more primordial version of the proteasome system (8). Although, in eukaryotes, 14 different genes encode for the core α and β subunits, in archaea, typically single α and β genes are responsible for the formation of homo-oligomeric rings that further assemble into the 20S proteasome; in archaea, a 19S particle is missing (9, 10). In certain bacterial species, mostly actinobacteria, proteasomes occur as well, which, like in archaea, tend to be composed of stacked homo-oligomeric α and β rings (11). The majority of bacterial species, however, lack classical proteasomes and contain other types of self-compartmentalizing proteases, such as HslV, a double-ring complex with 6-fold symmetry, and proteasome-unrelated cylindrical complexes like Lon or ClpP (12).

A major advantage of self-compartmentalizing proteases lies in the internalization of their active sites within the cylinders, thus minimizing uncontrolled proteolysis. The variety of large intracellular proteases in the different kingdoms of life is mirrored by the different solutions organisms have found to regulate access to these active sites. Proteasomes have added a layer of α subunits, providing gates that only open upon interaction with substrates or regulatory particles (3). Different types of AAA+^3^ interact with the core proteases, thereby serving multiple functions; they deliver substrate proteins, thus removing the recognition and specificity task in part from the protease itself; they unfold (mis)folded proteins to make them palatable to the protease; and they induce conformational changes in the protease, activating it and allowing translocation of unfolded substrates to the active sites at the inner cylinder surface (13). Not surprisingly, therefore, in extant organisms, most self-compartmentalizing proteases coexist and cooperate with such regulatory AAA+. The Rpt1–6 subunits cooperate in the context of the 19S lid with the eukaryotic proteasome. Vat and PAN are prominent cofactors of archaeal proteasomes, and the bacterial proteases HslV and ClpP have corresponding ring-shaped partner ATPases in the form of HslU and ClpX. In Lon, the AAA+ forms an integral domain of the complex (9, 13).

A couple of years ago, phylogenetic analysis revealed the existence of two uncharted bacterial members of the proteasome family, Anbu and BPH (14). Anbu is present in a phylogenetically diverse range of bacteria, and our bioinformatic sequence analysis put it at the evolutionary root of the proteasome system, where it may have shared a direct ancestry with the proto-β subunit in the last universal common ancestor (2). Interestingly, we could show that Anbu forms a helical open-ring structure that may be reminiscent of ancestral proteasome forms.

The second hitherto unknown bacterial proteasome family member was primarily found in Betaproteobacteria, hence its name BPH, short for Betaproteobacterial proteasome homologue (14). Virtually nothing is known about its structure and function. In this study, we present the first experimental characterization of two bacterial BPH proteins. Their crystal structures reveal homo-oligomeric double-barrel complexes with internalized active sites akin to those of proteasome and HslV. Unique structural features, however, like an elongated inner pore loop with a highly acidic sequence, distinguish BPH from its relatives. These features may compensate for the absence of regulatory α subunits and affect the BPH mechanism of action and its handling of substrates.

## Results and discussion

### BPH is an evolutionary descendant of HslV

**Figure 1.**
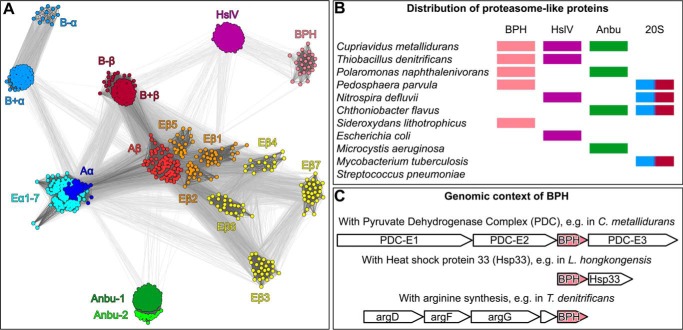
**Phylogeny and occurrence of BPH genes.** *A*, a cluster map of 969 proteasome-like sequences, with a maximum pairwise identity of 70%, was prepared using CLANS (15) based on their all-against-all pairwise similarities as measured by BLAST *p* values. Sequences are represented by *dots*, and the line coloring reflects BLAST *p* values (the darker a line is, the lower the *p* value). Proteasome subunits are abbreviated *E* for eukaryotic, *A* for archaeal, and *B*+/*B*− for Gram-positive and Gram-negative bacteria, respectively. *B*, the co-occurrence of BPH, HslV, Anbu, and the proteasome (20S) in representative organisms; *bars* are in the same color scheme as in *A. C*, genomic context of BPH in representative organisms.

To investigate the evolutionary history of BPH in the context of proteasome evolution, we searched for its homologs in the nonredundant protein sequence database and clustered them by pairwise sequence similarity. In contrast to standard phylogenetic methods, which work best with multiple alignments comprising at most a few thousand similar sequences, cluster analysis allows the analysis of very large datasets, comprising highly diverse sequences, as is the case for the proteasome family (15). The result is a map showing the interwoven relationships of the entire proteasome family. The center of this map (Fig. 1*A*) is dominated by α and β subunits from archaeal proteasomes from which all the other groups radiate. Eukaryotic α subunits group together with the archaeal ones in a large cluster, whereas the seven eukaryotic β subunits are more divergent and form distinct islands, with the catalytic subunits (β1, β2, and β5) being closest to the archaeal β subunits. Bacterial α and β subunits are organized into two separate subclusters, comprising subunits of Gram-positive actinobacteria that connect to the archaeal core clusters and subunits of the Gram-negative phyla, which may have acquired their proteasome by horizontal gene transfer from actinobacteria (16). Interestingly, bacterial Anbu exhibits only weak similarity to the bacterial α and β subunits but rather strong similarity to those of the archaeal proteasome and is supposed to have shared a direct ancestry with the proto-β subunit. The overall analysis of these sequence relationships led us to propose that the proteasome and Anbu were already present in the last universal common ancestor and that the proteasome was linearly inherited by archaea, bacteria, and eukaryotes (2). Where does this leave HslV and BPH? HslV sequences group into a single cluster, exhibiting equally significant similarity to actinobacterial and archaeal β subunits but only residual similarity to α subunits, suggesting that HslV arose from a proteasomal β subunit after the diversification of α and β, very early in bacterial evolution (2). Subsequently, it might have replaced the proteasome in many bacterial phyla. BPH sequences are positioned at the periphery of the map and are a more recent addition to the proteasome family. Confirming an initial hypothesis (14), our cluster analysis shows that BPH branches off from HslV and appears to be its direct descendant. Strictly speaking, however, the acronym BPH does not accurately reflect the distribution of this protein in bacterial species as, in variance with the initial description (14), BPH is also found outside the Betaproteobacteria, *e.g.* in members of Verrucomicrobia, Alpha- and Gammaproteobacteria, Cyanobacteria, or Acidobacteria (Fig. 1*B*).

### BPH genes are constitutively expressed genes occurring in stress-regulated operons

Before embarking on the biochemical characterization of BPH proteins, we tried to extract information about their physiological role from the genomic context of their genes. In prokaryotes, genes that functionally and/or physically interact are often grouped together in their respective genomes (17). For BPH genes, three types of operon environments were found to prevail (14) (Fig. 1*C*). In some organisms, *e.g. Thiobacillus denitrificans*, BPH is grouped in an operon with argininosuccinate synthase (argG) and adjacent to argD and argF, proteins involved in arginine biosynthesis. In other organisms, *e.g. Cupriavidus metallidurans*, BPH is found in an operon with the three genes encoding the pyruvate dehydrogenase complex (PDC). Finally, in other organisms, *e.g. Laribacter hongkongensis*, BPH is in the same operon as heat shock protein 33 (Hsp33). None of these operon contexts hint at the existence of regulatory partner proteins for BPH, nor of companion AAA that could act as unfoldases. Moreover, when we searched BPH-containing genomes for suitable ATPase candidates, none were found. To address the issue of interaction partners experimentally, we performed pulldown assays using cell extracts from *C. metallidurans* and differently tagged versions of BPH as bait. Pull-downs were done on HA, Strep, or Myc magnetic beads in the presence or absence of nucleotide (Mg-ATPγS) and also with a crosslinker to stabilize weak interactions. Candidate bands obtained on SDS gels were analyzed by mass spectrometry, but no obvious interaction partners could be identified (data not shown). Notably, neither PDC subunits (from the same operon) nor HslU (as a potential partner ATPase) were found to be associated with BPH.

Even though, at first glance, the three different operon contexts do not seem to have much in common, it turns out that they are all linked to the cellular stress response. Hsp33 is a chaperone expressed under heat shock conditions and activated under oxidative stress (18). Similarly, arginine biosynthesis is increased during heat shock and oxidative stress in several species (19–21). Arg is the substrate for nitric oxide synthase, whose product nitric oxide interferes with the formation of reduced thiols and the recycling of ferrous iron, thus diminishing the generation of damaging hydroxyl radicals (22). Finally, PDC subunits are prone to carbonylation, an irreversible oxidation of amino acid side chains caused by hydroxyl radicals (23), which, among many other proteins, inactivates PDC and turns it into a target for regulated degradation. These correlations suggested a protease function for BPH in the oxidative stress response, perhaps similar to the assumed function of the ATP-independent 20S proteasome (24). However, the carbonylation patterns of extracts from *C. metallidurans* grown under oxidative stress conditions or of *Escherichia coli* strain KY2266, in which the major cytosolic proteases are deleted (Δ*lon*, Δ*hslVU*, and Δ*clpPX*), did not change upon incubation with BPH (Fig. S1, *A* and *B*). Similar results were obtained upon oxidative stress treatment *in vivo* with KY2266 containing wildtype and mutant Cm-BPH forms (Figs. S1*C* and S2), suggesting that BPH cannot substitute for the missing self-compartmentalizing proteases.

### BPH assembles into tetradecameric double-ring complexes with a unique acidic inner surface

**Figure 2.**
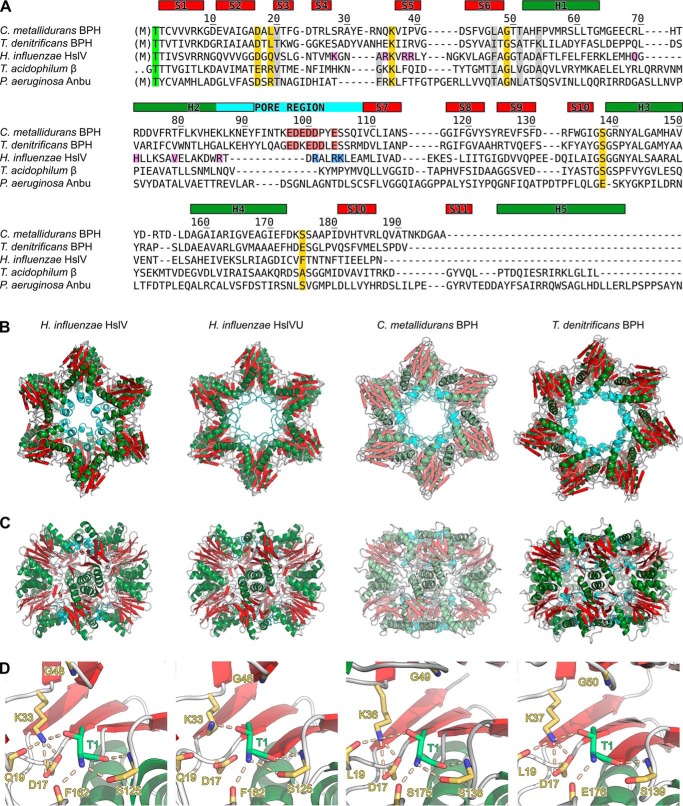
**Sequence and structure comparison of BPH and HslV complexes.** *A*, structure-based sequence alignment of proteasome-like proteins. Active site residues (*yellow*, Thr-1 in *green*), residues forming the substrate specificity pocket S1 (*gray*, Ref. 29), and HslU interface residues (*purple*) are highlighted. Residues accounting for the acidic BPH inner cavity characteristics are shown in *red* and the corresponding HslV Arg-86/Arg-89/Lys-90 basic cluster in *blue*. Secondary structure elements and the pore region are highlighted *red* (sheets), *green* (helices), or *cyan* (pore region); residue numbers correspond to *C. metallidurans* BPH. *B* and *C*, top and side view representations of *C. metallidurans* and *T. denitrificans* BPH crystal structures in comparison with *H. influenzae* HslV in the presence (PDB code 1G3I) or absence (PDB code 1G3K) of HslU. The non-native dodecameric Cm-BPH assembly is shown in *pale colors. D*, active site comparison, highlighting the catalytically important residues (Groll *et al.* (53)).

In the absence of obvious interaction partners, the question arises of how BPH recognizes its substrates. Moreover, how is access granted to the active sites unless they are not shielded? We tried to find answers to these questions in the molecular architecture of BPH and did a structural characterization of this novel protein complex. We opted for the analysis of BPH from *C. metallidurans* and *T. denitrificans*, two representatives of the Betaproteobacteria but with different lifestyles. *C. metallidurans* is an aerobic chemolithoautotroph that degrades xenobiotics and is adapted to survive heavy metal stress (25), whereas *T. denitrificans* is a facultatively anaerobic chemolithoautotroph oxidizing inorganic sulfur compounds (26). Both BPH sequences are shown in the alignment in Fig. 2*A*. All active-site residues (*yellow*) are conserved in BPH proteins, with the noticeable presence of Ser as an N-terminal nucleophile in BPH-containing organisms outside of the Betaproteobacteria (Fig. S3). The BPH sequences do not feature propeptides, suggesting that, contrary to the situation in the proteasome, the subunits should be in their active state right after assembly. As one would predict, residues mediating the interaction of HslV with HslU (Fig. 2*A*, *purple*) are not conserved in its descendant BPH, which became independent of the accessory ATPase.

**Figure 3.**
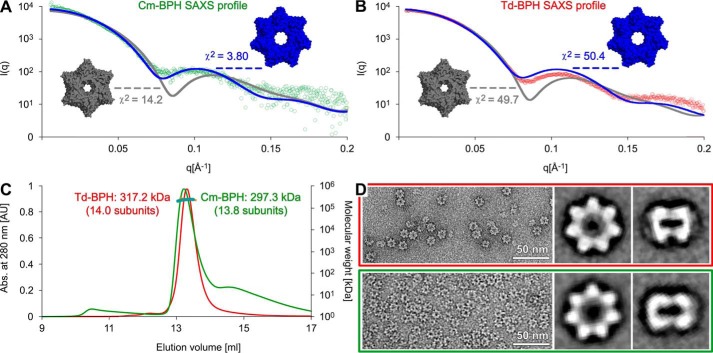
**BPH forms a tetradecameric double ring in solution.** *A* and *B*, experimental SAXS data are plotted together with the theoretical profiles and χ^2^ values of dodecameric (*gray*) or tetradecameric ring structures (*blue*). *C*, SEC-MALS profiles for Cm-BPH (*green*) and Td-BPH (*red*). Absorbance is measured in arbitrary units (*AU*). *D*, electron micrographs of Td-BPH (*red box*) and Cm-BPH (*green box*). Particles were classified and averaged, resulting in top view (*center*) and side view (*right*) representations of both proteins.

When recombinantly expressed in *E. coli*, both Cm-BPH and Td-BPH formed stable complexes (*T_m_* 63 °C and 61 °C, respectively) that eluted early in size exclusion chromatography (SEC), as expected for dodecameric or tetradecameric complexes. In crystallization trials, we obtained for Td-BPH a hexagonal crystal form diffracting to about 2.7 Å and an orthorhombic one diffracting to 2.3 Å. We could solve the structure of the hexagonal crystal form in space group P6_1_22 in a single anomalous dispersion (SAD) approach using a selenomethionine derivative. The crystals contain a heptameric ring of Td-BPH protomers in the asymmetric unit (ASU), which represents one half-of a tetradecameric double ring constructed by crystallographic 2-fold symmetry. The higher-resolution orthorhombic crystal form could subsequently be solved in space group P2_1_2_1_2_1_ via molecular replacement, locating a single tetradecameric double ring in the ASU. The tetradecameric complexes from both crystal forms are virtually identical, and most of the sequence is resolved in all chains from Thr1 until Pro-190. However, in both crystal forms, the center portion of the pore loop (between Gln-95 and Glu-101) is disordered. In contrast to the Td-BPH crystals, the obtained Cm-BPH crystal form was not compatible with an assembly of 7-fold symmetry. The data could be scaled to 2.1 Å in space group P6, with unit cell dimensions suggesting two monomers in the ASU. Searching with a single Td-BPH protomer, molecular replacement successfully located the expected two monomers in the ASU. Via the 6-fold symmetry, they form a dodecameric double ring with intersubunit interfaces reminiscent of the tetradecameric Td-BPH but resembling the architecture of dodecameric HslV. One way to explain this difference is to invoke structural diversity within the BPH family; after all, both 6-fold (HslV) and 7-fold (proteasome) symmetries persist in related proteins of the same fold. However, another possible explanation is a crystallization artifact for one of the proteins. To address this issue, we subjected Cm-BPH and Td-BPH to biophysical characterization, including analytical ultracentrifugation, SEC-coupled multiangle light scattering (SEC-MALS), small angle X-ray scattering (SEC-SAXS), and electron microscopy. In analytical ultracentrifugation and SEC-MALS runs, both proteins behaved almost identically, yielding s_20,w_ sedimentation coefficients of 12.01 and 12.08 and molecular masses of 297.3 kDa (13.8 subunits) and 317.2 kDa (14.0 subunits) for Cm-BPH and Td-BPH, respectively (Fig. S4 and Fig. 3*C*). Also, in the SEC-SAXS experiments, the profiles of both proteins were very similar. We fit each profile with a dodecameric (from the Cm-BPH crystal structure) and a tetradecameric model (from the Td-BPH crystal structure). For both proteins, the tetradecameric model convincingly reproduced the features of the SAXS profile in the low-q range < 0.15 Å^−1^, in contrast to the dodecameric model. For Cm-BPH, the fit of the tetrameric model also resulted in a significantly lower χ^2^ value of 3.80, ruling out the dodecamer with χ^2^ = 14.2 (Fig. 3*A*). Notably, these fits were obtained without any conformational refinement to relax the barrel geometries from potential crystal packing restraints and without prior modeling to address sequence conflicts between the two proteins. Finally, when we averaged negative-stain electron micrographs of Cm-BPH and Td-BPH particles, exclusively seven-membered rings were obtained for both proteins (Fig. 3*D*). In summary, both BPH proteins unequivocally form double rings with 7-fold symmetry in solution.

Why then could we obtain crystals of Cm-BPH as a dodecamer? A closer inspection of the Cm-BPH crystal structure reveals two malonate molecules from the crystallization buffer to be bound per Cm-BPH protomer, specifically intercalated into the intersubunit clefts of the rings from the outside (Fig. S5). Like wedges, they bridge a part of the native intersubunit interactions formed in the Td-BPH rings, thereby leading to an increased curvature favoring hexameric rings. These hexamers are presumably stabilized in the crystallization process, allowing for the tight crystal packing depicted in Fig. S5. A potential result of the smaller radius is a different conformation of the pore loop as compared with the Td-BPH structure. The smaller radius allows for a tighter interaction between neighboring pore loops within the rings. In contrast to Td-BPH, the pore loops are fully ordered, although this presumably represents a non-physiological conformation. Although unmasked as artificial, the 6-fold symmetric crystal structure of Cm-BPH bears interesting evolutionary clues. In the course of its evolution from the 6-fold-symmetric HslV, BPH had to retune its geometry for 7-fold symmetry. The apparent ability of Cm-BPH to assemble into closed rings of ancestral symmetry, albeit artificially stabilized by intercalating malonate molecules in a rigid crystal packing, demonstrates how small structural changes (mimicked by the malonate) can affect the oligomerization symmetry within the proteasome family.

**Figure 4.**
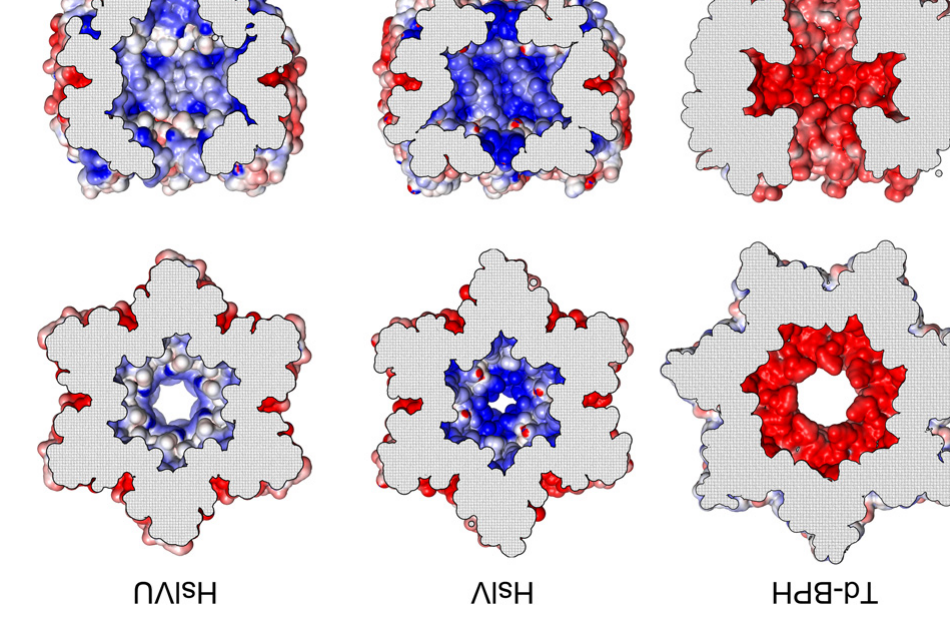
**Comparison of BPH and HslV inner cavity characteristics.** Top and side views of cut-open rings from Td-BPH, *H. influenzae* HslV (PDB code 1G3K) and *H. influenzae* HslV in the presence of HslU (HslVU, PDB code 1G3I) are shown with Poisson–Boltzmann electrostatic potentials (± 5 *kT/e*) plotted on the surface (52). Negative potentials are visualized in *blue* and positive potentials in *red*.

If one looks at the inner dimensions of various proteasome-like barrels, it is evident that BPH displays somewhat larger pores inside the cylinder in comparison with HslV. In the presence of HslU, however, the HslV pore is known to widen considerably (Fig. 2, *B* and *C*). Taking a closer look at the inner cavity, one finds that BPH proteins are distinguished from other proteasomal proteins by their rather long pore loops. Although, in HslV, the loop is merely a short linker between H2 and S7, the extended BPH pore loop is long enough to make contacts with its adjacent subunit (Fig. 2, *A–C*). Via their the N- and C-terminal ends, the pore loops of adjacent subunits form short β strands lining the pore surface. Moreover, as BPH pore loops have a very acidic sequence lineup, exemplified by five successive Asp/Glu residues in Cm-BPH, this results in a unique inner surface environment (Fig. 4) that is in striking contrast to that of its ancestor HslV, where the basic residues Arg-86, Arg-89, and K-90 protrude toward the inner cavity at the pore entrance. Interestingly, point mutations in these residues cause a severe decrease of the proteolytic activity of HslV (27), and HslU binding induces structural changes in this region, resulting in a switch of cavity characteristics (Fig. 4).

### Active-site characteristics of BPH: Serine as an alternative catalytic residue

**Figure 5.**
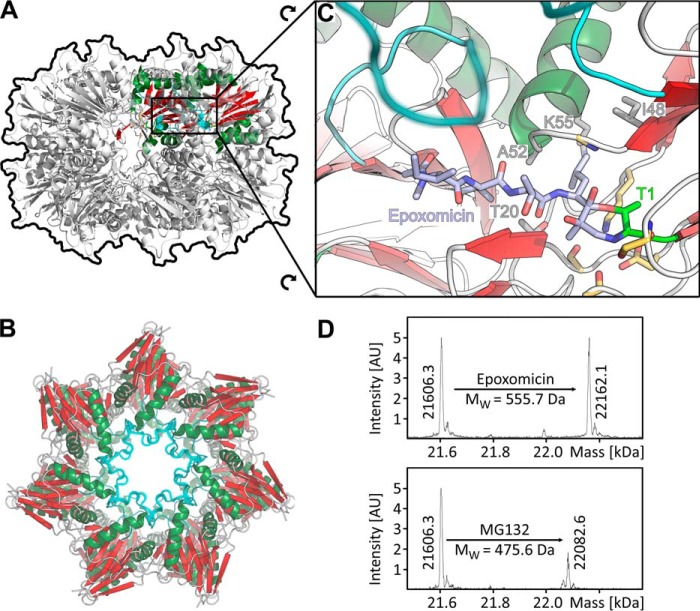
**Binding of the proteasome-specific inhibitor epoxomicin to the Td-BPH active site is accompanied by structural transitions in the pore loop.** *A* and *B*, top and side view representation of epoxomicin-conjugated Td-BPH. In contrast to the unconjugated structure (Fig. 2), a mostly continuous electron density allows to approximately trace the pore loop backbone (*blurred*). *C*, close-up view of the Td-BPH active site, with catalytic nucleophile Thr1 (*green*), residues thought to be crucial for catalysis (*yellow*), and residues forming the substrate specificity pocket S1 (*gray*) represented as *sticks*. The active site Thr-1 is inhibited through formation of a morpholino adduct, as described for the proteasome (34). The Leu side chain N-terminal of the reactive epoxyketone moiety is coordinated by the Td-BPH S1 pocket residues. The middle of the pore loop (*cyan*) becomes apparent in approximate van der Waals distance closed over the peptide-like moiety, suggesting a role in substrate recognition. *D*, epoxomicin and MG132 modify the Cm-BPH active site. Cm-BPH was treated with the proteasome-specific inhibitors MG132 or epoxomicin and analyzed by mass spectrometry. The shifts in protomer masses correspond exactly to the molecular masses of the respective inhibitors. The mass of the unconjugated form (21606.3) shows that the start Met was removed to expose the catalytic Thr-1 amino group.

The prototypical BPH active sites display all the features that are characteristic for proteasome-like proteins. Prominent residues in Td-BPH are Thr-1, Asp-17, and Lys-37 (Fig. 2*D*), with Thr-1 acting as catalytic nucleophile. Lys-37 corresponds to Lys-33 in the proteasome; its function is to facilitate deprotonation of Thr-1 (28). Asp-17 assists in this reaction by orienting Lys-37 and making it prone to protonation. Equally present are *e.g.* conserved Gly-50, which helps to provide the oxyanion hole that stabilizes the transition state, and Ser-139, which forms hydrogen bonds with Thr-1. The S1 pocket, determining substrate specificity (29), is of hydrophobic character, priming it for the accommodation of uncharged amino acids. An interesting and highly unusual feature is the presence of Ser as the N-terminal residue in all BPH proteins outside the Betaproteobacteria (Fig. S3). Aside from that peculiarity, the other active-site residues in these cases conform to the typical proteasome family pattern (Fig. 2*D*). To our knowledge, apart from the aforementioned BPH proteins, Ser occurs in only two proteasome family members as the catalytic residue, one Anbu representative (Amb0901 from *Magnetospirillum magneticum AMB-1*), and one HslV protein (CodW from *Bacillus subtilis* (30)), which is active only in the presence of its partner ATPase HslU (CodX) (30). In enzymatic activity assays using a wide range of standard substrate peptides and the intrinsically disordered protein casein, we found Cm-BPH and Td-BPH to be inert (data not shown), even though mass spectrometry (Fig. 5*D*) and the crystal structures (Fig. 2, *B* and *C*) show that Thr-1 is exposed and not modified. Unlike with the 20S proteasome, which typically exists in a latent form that must be tweaked with SDS to show significant activity in these assays (31, 32), addition of the detergent had no effect on BPH. The apparent lack of protease activity could be explained by the need for an as yet unknown cofactor, required to allosterically activate BPH or to deliver substrate proteins in a conformation that we do not mimic *in vitro*. In addition, BPH might be less promiscuous than the housekeeping proteases, with a specialized substrate spectrum.

To ascertain the functional nature of the BPH active site, we covalently modified it with the proteasome-specific inhibitor epoxomicin. This compound features an epoxyketone electrophilic trap, the warhead, which renders it highly selective toward the catalytic Thr-1 of proteasome-like proteins (33). The ligand-binding affinity resides in a peptide-like moiety, resembling the sequence Ile-Ile-Thr-Leu. When we incubated Cm-BPH with epoxomicin or the inhibitor MG132, we readily obtained modified protein, as seen in mass spectra (Fig. 5*D*). Additionally, we obtained co-crystals of Td-BPH with bound epoxomicin diffracting to 2.95 Å, whose structure could be solved on the basis of the apo Td-BPH coordinates (Fig. 5, *A–C*). Epoxomicin is bound in all subunits, in the same fashion as in comparable co-structures with epoxomicin, which are only available for the proteasome but not for HslV or Anbu (34). The structure is consistent with productive binding of the inhibitor. The peptide-like moiety lines up in the substrate binding pocket to form an additional β-strand to the S3–S4 β hairpin, as in the proteasome (29). In particular, the modification of Td-BPH Thr-1 by the epoxomicin warhead confirms this residue as the active nucleophile in the catalytic reaction cycle. Thus, all prerequisites for functionality are met by the layout of the BPH active site. Further, an interesting peculiarity of the inhibitor-bound structure concerns the pore loop. Although the central portion of this loop is unresolved in the ligand-free Td-BPH structure, a mostly continuous electron density is observed for the whole loop in the bound structure, allowing to approximately trace its backbone (Fig. 5*C*). The center portion of the loop is closed over the epoxomicin peptide-like moiety. It is therefore conceivable that the loop contributes to substrate recognition, adopting a well-defined conformation and forming specific interactions with yet to be identified native substrates. Conversely, it seemed possible that the pore loops of our BPH proteins were incompatible with the model substrates of our activity assays (Fig. S6). Consequently, we sought to obtain versions with different pore properties. However, when we replaced the acidic loops in Cm-BPH and Td-BPH with small uncharged amino acid residues, the BPH mutant proteins still assembled into stable double-ring complexes but showed no activity (data not shown). This leaves the contribution of the pore loop to substrate recognition and gating an important open question to explore in the future.

### Conclusions

Our structural analysis of BPH proteins has revealed the formation of homo-oligomeric barrels with active sites at the inside of the cavity. Despite their descendance from dodecameric HslV, BPH proteins have acquired a 7-fold symmetry like the proteasome, indicating inherent versatility of the proteasome fold to undergo symmetry rearrangements and to form different types of assemblies in the course of their evolution. It is plausible that BPH originated from HslV before the emergence of Betaproteobacteria, which still seem to provide a suitable ecological niche for this protease, whereas, in many other bacterial species, the gene disappeared over time. In extant organisms, BPH would be a vestige of the past that survived because of a beneficial function or activity. As there is a fair amount of redundancy among bacterial cytosolic proteases, such an activity would not have to be unique but could be overlapping with that of its evolutionary relatives. In fact, BPH occurs in bacterial species in various combinations together with Anbu, HslV, or the 20S proteasome or is even the sole proteasome family member present, *e.g.* in *Sideroxydans lithotrophicus* (Fig. 1*B*). Its organismal distribution does not correlate with that of its ancestor HslV, in line with the notion that HslV was also lost from a number of genomes at later times. The phylogenetically widespread although sparse occurrence of BPH outside the Betaproteobacteria could be attributed to horizontal gene transfer. Arguing against this is the finding that all BPH sequences outside the Betaproteobacteria feature Ser-1 as the active-site residue (Fig. S3), in contrast to the prototypical Thr-1 (Fig. 2*A*), rather suggesting that, already early on, different BPH variants existed that then diverged in their evolutionary paths. The presence of Ser-1 is peculiar, as it was shown for the proteasome that this residue is less efficient than Thr-1, which was ascribed to an unfavorable orientation of Ser-1 toward incoming substrates (28). Early in descendance from HslV, the initial co-occurrence in the same cell might have made it necessary to structurally distinguish BPH and to keep HslU interaction reserved for HslV. This would have allowed BPH to move toward ATP-independent functions, for which HslV seems to have little or no propensity (35, 36). The evolution of the distinct acidic pore loop of BPH with its potential role in substrate recognition can be seen as part of these mechanistic and structural changes. We may miss ATP-independent functions in the universally used standard activity assays, which do not comprehensively cover all possible binding and cleavage specificities. In this context, it is noteworthy that the protease ClpP exhibits remarkable specificity for certain types of fluorescent peptides *in vitro*, which is not mirrored in the proteolysis of endogenous substrates *in vivo*, where ClpP seems to be more promiscuous (37). Effective local concentrations of active sites and substrate in the protease barrel reaching the high millimolar range have been proposed to cause this relaxed cleavage specificity (37). This could also apply to BPH and Anbu, for which no *in vitro* activity has been obtained either (2). Consequently, future studies will have to focus on the identification of the physiological substrate spectrum of BPH.

## Experimental procedures

### Bioinformatics and phylogenetic analysis

To gather sequences of proteasome homologs, the non-redundant protein sequence database at the NCBI, comprising either bacterial, archaeal, or eukaryotic proteins, was searched with four iterations of PSI-BLAST at default settings (38). The following proteins were used as seeds for the first iteration of these searches: *Pseudomonas aeruginosa* Anbu, *Haemophilus influenzae* HslV, *C. metallidurans* BPH, *Thermoplasma acidophilum*, and *Mycobacterium tuberculosis* proteasome α and β. After each iteration, sequences to be included for the next iteration were manually reviewed. The sequences resulting from each of the searches were filtered down to a pairwise sequence identity of 90% using HHfilter in the MPI Bioinformatics Toolkit (39). The sequences in these reduced sets were next clustered by their all-against-all pairwise BLAST *p* values in CLANS (15) to identify and remove incomplete or unrelated sequences. Sequences contained in the individual clusters of the resulting cluster maps were subsequently aligned using PROMALS3D (40), based on homologs with three-dimensional structures. The alignments were manually refined, and propeptides as well as inserts of unusual lengths were removed. To further decrease redundancy, for the purpose of creating a global cluster map of proteasome homologs, these alignments were filtered down to a maximum pairwise identity of 70% using HHfilter. Next, all sequences in these alignments were pooled and clustered in CLANS to generate the cluster map of proteasome homologs shown in Fig. 1*A*. Clustering was done to equilibrium in 2D at a BLAST *p* value cutoff of 1e-20 and the final cluster map was made by showing all connections with a *p* value better than 1e-09. The alignment shown in Fig. 2 was prepared based on the indicated crystal structures with PROMALS3D (40) and manually refined.

### Cloning, protein expression, and purification

*C. metallidurans* CH34 (DSM 2839) and *T. denitrificans* AB7 (DSM 12475) genomic DNA were used to amplify and clone the genes Rmet_1198 (Cm-BPH) and B059_01700 (corresponding to *T. denitrificans* ATCC 25259 gene Tbd_1847) (Td-BPH) into pET22b and pET30b expression vectors using NdeI and HindIII restriction sites. The constructs for the pET22b vector were extended via PCR with C-terminal tobacco etch virus cleavage motifs followed by His_6_ tags. The constructs for the pET30b vector were extended with C-terminal Strep, Myc, and HA tags or used without a protein tag. Acidic pore loop mutants of Cm-BPH (E97A, D98G, E99A, D100G, and D101A) and Td-BPH (E98A, D99G, K100A, E101G, and D102A) were generated by site-directed mutagenesis (41). Wild-type and Trap Cm-BPH mutant (T1A, T2A, and C3A) genes were also cloned into pBAD/Myc-His vectors using XhoI and HindIII restriction sites. *E. coli* BL21 gold or KY2266 (42) cells (Thermo) were transformed with the respective plasmids and grown at 25 °C in M9 minimal medium supplemented with 50 μg/ml Se-Met, Leu, Ile, Phe, Thr, Lys, and Val for Se-Met labeling or in lysogeny broth for all other purposes. Protein expression was induced at an optical density of 0.4 at 600 nm with 0.5 mm isopropyl-β-d-thiogalactoside (BL21 gold) or 1% arabinose (pBAD vector constructs in KY2266). Cells were harvested after 16 h, lysed by French press, and cleared from cell debris by ultracentrifugation. Soluble proteins were purified via two successive anion exchange steps with HiTrap Q HP (20 mm MES-NaOH (pH 6.2), 1 mm DTT, and 50–500 mm NaCl) and MonoQ HR 16/10 (20 mm Tris-HCl (pH 8.5) and 50–500 mm NaCl) columns or via HisTrap HP columns (20 mm Tris-HCl (pH 8.0), 250 mm NaCl, and 20–250 mm imidazole). His_6_ tags were removed by incubation with 0.1 molar equivalents of His_6_–tobacco etch virus protease for 16 h at 4 °C, followed by another HisTrap HP purification step. Finally, all proteins were applied to a gel size exclusion chromatography (Sephacryl S-300 HR, GE Healthcare) using buffer A (20 mm HEPES-NaOH and 100 mm NaCl). Purified proteins were supplemented with 15% glycerol, flash-frozen in liquid nitrogen, and stored at −80 °C.

### Biochemical and cell biological techniques

Proteolytic activity was assayed in buffer A supplemented with complete protease inhibitor (Roche) without EDTA, which does not inhibit proteasome-like proteases. Enzyme (10 nm subunits when concentration was fixed) and fluorogenic substrates (50 μm each) were incubated at 30 °C, and fluorescence changes were recorded continuously for 2 h (Synergy H4 microplate reader, Biotek). Assay parameters were modified by varying pH (4.5–9.0), temperature (25–60 °C), salt (50–500 mm NaCl, 0–50 mm KCl, and 0–5 mm MgCl_2_/CoCl_2_/CaCl_2_), SDS (0–0.1%), and enzyme concentrations (1–1000 nm). Assayed substrates included BODIPY–casein (Thermo), Ac-Gly-Pro-Leu-Asp-AMC, Z-Leu-Leu-Glu-AMC, Suc-Leu-Leu-Val-Tyr-AMC, Ac-Arg-Leu-Arg-AMC, Boc-Leu-Arg-Arg-AMC, and Z-Gly-Gly-Leu-AMC (Enzo Life Sciences); Mca-Ala-Lys-Val-Tyr-Pro-Tyr-Pro-Met-Glu-Dap(Dnp) (GenScript); H-Val-AMC, H-Tyr-AMC, H-Thr-AMC, H-Pro-AMC, H-Phe-AMC, H-Asp-AMC, H-Ala-AMC, and H-Ile-AMC (Bachem); and the P-check peptide library (Jena Bioscience).

To identify BPH interactors, C-terminally HA-, Myc-, or Strep-tagged Cm-BPH proteins were produced in *E. coli* and bound to streptavidin-, anti-HA-, or anti-Myc-coated magnetic beads in buffer B (25 mm HEPES-NaOH 7.2, 130 mm NaCl, 10 mm KCl, 0.1% Nonidet P-40, and 1× complete protease inhibitor (Roche)). Beads were then incubated with *C. metallidurans* mid-log phase extracts at 4 °C for 12 h (buffer B without Nonidet P-40 but with DNase and 1 mm MgCl_2_) in the presence or absence of 1 mm Mg^2+^-ATPγS. After six washes with buffer B, BPH and bound proteins were eluted with either 2 mg/ml HA or Myc peptides or 2.5 mm desthiobiotin and analyzed by SDS-PAGE followed by mass spectrometry. In a second experiment, purified Strep-tagged Cm-BPH proteins were cross-linked at 25 °C to potential interactors in *C. metallidurans* extract by addition of either 25 mm formaldehyde for 30 min or 50 mm for 60 min. Reactions were stopped by addition of a 4-fold excess of glycine, and the pull-down was performed and analyzed as described above.

The *in vivo* effect of BPH expression on H_2_O_2_ susceptibility was studied in the *E. coli* mutant strain KY2266 defective in cytosolic proteases (Δ*lon*, Δ*hslVU*, and Δ*clpPX*). Log-phase cells expressing either wildtype or trap mutant Cm-BPH were diluted to optical density 0.1, and the medium (lysogeny broth and 1% arabinose) was supplemented with the indicated H_2_O_2_ concentrations. Growth curves were recorded in three independent experiments for 4 h at 30 °C and compared with those of KY2266 without plasmid and wildtype MC4100 cells.

The concentration of carbonyl groups in oxidized proteins was quantified photometrically with 2,4-dinitrophenylhydrazine (DNPH) (43), using cell extracts. Oxidative stress was brought about by prolonged 16-h stationary phase incubation at 30 °C in the presence or absence of 10 μm MG132. The resulting lysate was separated from cell debris via ultracentrifugation and incubated with 12.4 μm DNPH in the presence of 2 m HCl for 15 min at 25 °C. Modified proteins were precipitated with 25% trichloroacetic acid, separated from residual DNPH by three successive washes, and resuspended in 6 m guanidine and 100 mm Tris-HCl (pH 8.8). The relative concentration of modified carbonyl groups was quantified by measuring the absorbance of the samples at both 370 nm (DNPH) and 280 nm (protein). The numbers shown in Fig. S1 represent mean values determined in at least three independent experiments.

### Biophysical methods

Thermal denaturation curves to monitor protein stability were recorded by circular dichroism spectroscopy at 220 nm using a JASCO J-810 spectropolarimeter. For EM, glow-discharged carbon-coated grids were incubated with 0.1 mg/ml protein suspension, stained with 1% uranyl acetate, and examined with a FEI Tecnai G2 Spirit BioTwin transmission EM at 120 kV. Images were collected on a Gatan Ultrascan 4000 camera. Particles were selected manually, and image processing was carried out using EMAN2 (44).

Sedimentation velocity experiments were carried out in a Beckman Coulter Optima XL-I analytical ultracentrifuge using both interference and absorbance detection. Experiments were performed with 0.5 mg/ml protein in buffer A at 20 °C, an angular velocity of 40,000 rpm was applied. Samples were filled in titanium cuvettes with an optical pathlength of 20 mm. Solvent density and viscosity were calculated according to the buffer composition with Sednterp v.2. The partial specific volumes of Cm-BPH (0.729 ml/g) and Td-BPH (0.732 ml/g) were incrementally calculated from the amino acid sequence. Evaluation was carried out as global fitting to approximate solutions of the Lamm equation with Sedfit v.14.6. The frictional properties of the molecules in terms of the frictional ratio f/f0 were treated as floating parameters.

For LC-MS measurements, 1 mg/ml Cm-BPH or Td-BPH was incubated with either a 20× molar excess of epoxomicin or a 2× molar excess of MG132 for 48 h at 4 °C in buffer A. To determine the masses of BPH protomers, desalted samples were subjected to a Phenomenex Aeris Widepore 3.6 μm C4 200 Å (100 × 2.1 mm) column using an Agilent 1100 HPLC, eluted with a 30–80% H_2_O/acetonitrile gradient over 15 min at a flow rate of 0.25 ml/min in the presence of 0.05% trifluoroacetic acid, and analyzed with a Bruker Daltonik microTOF. Eluted proteins were ionized at 4500 V and mass-to-charge (*m*/*z*) ratios were determined in the range of 800–3000. Data processing was performed in Compass DataAnalysis 4.2, and the *m*/*z* was deconvoluted to obtain the protein mass via MaxEntropie.

SAXS experiments were performed at beamline B21, Diamond Light Source (Didcot, UK), with an X-ray wavelength of 1 Å and a Pilatus 2 m detector at a distance of 3.9 m. Samples of 50 μl of Cm-BPH and Td-BPH at concentrations of 8.25 mg/ml and 15 mg/ml were delivered at 20 °C via an in-line Agilent HPLC with a Shodex Kw-403 column and a running buffer consisting of 20 mm HEPES-NaOH (pH 8.0), 150 mm NaCl, 2 mm tris(2-carboxyethyl)phosphine, and 1% sucrose. The continuously eluting samples were exposed for 300 s in 10-s acquisition blocks, and the data were preprocessed using in-house software. Frames recorded immediately before elution of the sample were used for buffer subtraction. Buffer subtraction and further analysis were performed with ScÅtter version 2.2b. The crystallographic models of dodecameric Cm-BPH and tetradecameric Td-BPH were fit to both resulting SAXS profiles using the program FoXS (45).

### Crystallization, data collection, and structure determination

Cm-BPH and Td-BPH were concentrated to 11.2 and 20 mg/ml, respectively, in 50 mm NaCl and 20 mm Tris-HCl (pH 7.5). Screening of conditions was performed in 96-well sitting-drop plates, with drops containing 300 nl of protein solution and 300 nl of reservoir solution and a reservoir of 50 μl. The crystals used in the diffraction experiments grew within 2 days in 100 mm sodium acetate (pH 4.4), 1.5 m sodium nitrate for Cm-BPH, 100 mm sodium acetate (pH 4.8), 1.2 m sodium nitrate for the hexagonal Td-BPH crystal form, and 100 mm HEPES-NaOH (pH 7.0), 600 mm NaF for the orthorhombic Td-BPH crystal form. For SAD phasing, the hexagonal Td-BPH crystals were reproduced using a Se-Met derivative. Td-BPH:epoxomicin co-crystals grew under this same condition, with 1 mg/ml of epoxomicin added to the protein solution. Prior to loop-mounting and flash-cooling in liquid nitrogen, the Cm-BPH crystals and all Td-BPH crystals of the hexagonal crystal form were briefly transferred into a droplet of reservoir solution supplemented with 30% ethylene glycol for cryoprotection. Data were collected at 100 K at beamline X10SA of the Swiss Light Source (Villigen, Switzerland) using a Pilatus 6 M-F hybrid pixel detector (Dectris Ltd.). All data were indexed, integrated, and scaled using XDS (46), with the statistics given in Table S1.

For SAD phasing of the hexagonal Td-BPH crystal form in space group P6_1_22, we employed SHELXD (47) for heavy atom location, finding 28 selenium sites belonging to seven chains in the ASU. After phasing and density modification with SHELXE (47), most of the structure could be traced and built by Buccaneer (48). The Td-BPH:epoxomicin co-structure and the orthorhombic crystal form in space group P2_1_2_1_2_1_ were subsequently solved on the basis of the P6_1_22 coordinates. As there were no conformational differences detectable between the apo structures in P6_1_22 and P2_1_2_1_2_1_, refinement was continued and finalized with the higher-resolution P2_1_2_1_2_1_ data. The structure of Cm-BPH was solved by molecular replacement with MOLREP (49), using a single subunit of the Td-BPH structure as the search model, locating two subunits in the ASU. The sequence of the chains was retraced using Buccaneer (48). All structures were completed by cyclic manual modeling with Coot (50) and refinement with REFMAC5 (51), resulting in the refinement statistics given in Table S1. The structures were deposited in the PDB under accession codes 5OVS (Td-BPH), 5OVT (Td-BPH:epoxomicin), and 5OVU (Cm-BPH). Structures were visualized using PyMOL v1.8.0.5, and electrostatic potentials were calculated at ± 5 *kT/e* (at 298K 1*kT/e* equals 25.7 mV) with the APBS plugin and PDB code PDB2PQR using default settings (52).

## Author contributions

A. C. D. F., M. D. H., and J. M. designed the experiments. A. C. D. F., L. M., K. H., and M. D. H. performed the experiments. A. C. D. F., K. H., M. D. H., and J. M. analyzed the data. A. C. D. F., M. D. H., and J. M. wrote the manuscript; and all authors reviewed the results and approved the final version of the manuscript.

## Supplementary Material

Supporting Information

## Footnotes

3 The abbreviations used are:

AAA: ATPase associated with diverse cellular activities
BPH: Betaproteobacteria proteasome homolog
PDC: pyruvate dehydrogenase complex
ATPγS: adenosine 5′-*O*-(thiotriphosphate)
Cm: *Cupriavidus metallidurans*
Tm: *Thiobacillus denitrificans*
SEC: size exclusion chromatography
SAD: single anomalous dispersion
ASU: asymmetric unit
MALS: multiangle light scattering
SAXS: small-angle X-ray scattering
Se-Met: selenomethionine
AMC: 7-amino-4-methylcoumarin
DNPH: 2,4-dinitrophenylhydrazine.
