# Supplementary material for: Structural characterization of the bacterial proteasome homolog BPH reveals a tetradecameric double-ring complex with unique inner cavity properties
Source: J Biol Chem. 2017 Nov 28;293(3):920–30. doi: 10.1074/jbc.M117.815258 (PMC5777263; doi:10.1074/jbc.M117.815258)
Supplement: Supporting Information [file supp_293_3_920__index.html]

Structural characterization of the bacterial proteasome homolog BPH reveals a tetradecameric double-ring complex with unique inner cavity properties — Structural characterization of the bacterial proteasome homolog BPH reveals a tetradecameric double-ring complex with unique inner cavity properties — Structure of the novel bacterial proteasome homolog BPH — Supporting Information 

# Structural characterization of the bacterial proteasome homolog BPH reveals a tetradecameric double-ring complex with unique inner cavity properties

## Supporting Information

- Supplement (.pdf, 1.2 MB) - Supplement
