# Supplementary material for: Structural characterization of the bacterial proteasome homolog BPH reveals a tetradecameric double-ring complex with unique inner cavity properties
Source: J Biol Chem. 2017 Nov 28;293(3):920–30. doi: 10.1074/jbc.M117.815258 (PMC5777263; doi:10.1074/jbc.M117.815258)
Supplement: Supporting Information [file 10.1074_M117.815258_jbc.M117.815258-1.pdf]

Structural characterization of the bacterial proteasome homolog BPH reveals a tetradecameric double-ring complex with unique inner cavity properties

Adrian C. D. Fuchs[1], Lorena Maldoner[1], Katharina Hipp[2], Marcus D. Hartmann[1] and Jörg Martin[1]

[1]From the Department of Protein Evolution and the [2]Electron microscopy facility of the Max Planck Institute for Developmental Biology, Spemannstraße 35, 72076 Tübingen, Germany

Supplemental material included:

Table S1

Figures S1-S6

	Td-BPH SeMet	Td-BPH $P2_12_12_1$	Td-BPH - Epoxomicin	Cm-BPH
**Data collection**				
Space group	$P6_122$	$P2_12_12_1$	$P6_122$	P6
Cell dimensions a, *b*, *c* (Å)	197.3, 197.3, 297.2	79.1, 175.2, 205.9	196.8, 196.8, 296.4	96.5, 96.5, 75.9
Wavelength (Å)	0.978	1.0	1.0	1.0
Resolution (Å)	39.9 – 2.69 (2.86 – 2.69)	39.6 – 2.30 (2.44 – 2.30)	39.2 – 2.95 (3.12 – 2.95)	37.9 – 2.10 (2.22 – 2.10)
R_{merge}	29.0 (126.7)	11.3 (83.1)	43.9 (198.1)	14.4 (112.1)
$I/\sigma I$	26.6 (6.16)	11.7 (1.98)	13.6 (3.11)	11.7 (2.03)
$CC_{1/2}$ (%)	99.9 (94.7)	99.7 (70.4)	99.5 (87.8)	99.7 (79.6)
Completeness (%)	99.8 (98.9)	99.7 (98.7)	99.9 (100)	99.8 (99.3)
Redundancy	105.9 (97.7)	5.22 (5.14)	57.1 (57.6)	9.39 (9.33)
**Refinement**				
Resolution (Å)		39.6 – 2.30 (2.36 – 2.30)	39.2 – 2.95 (3.03 – 2.95)	37.9 – 2.10 (2.15 – 2.10)
No. reflections		121199	68023	22444
R_{work}		0.21 (0.35)	0.17 (0.32)	0.24 (0.35)
R_{free}		0.26 (0.39)	0.19 (0.35)	0.25 (0.34)
**PDB code**		5OVS	5OVT	5OVU

Table S1. Data collection and refinement statistics. Highest resolution shells are shown in parenthesis.

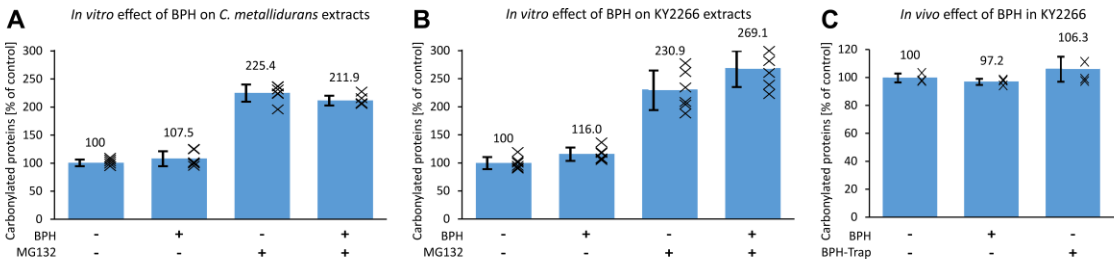

Figure S1. Effect of BPH on the levels of carbonylated proteins under oxidative stress. *A*,*B C. metallidurans* (*A*) or *E. coli* KY2266 (Δ*lon,* Δ*hslVU,* Δ*clpPX* (1)) *(B)* extracts were produced from stationary-phase cultures grown with or without the proteasome inhibitor MG132 and treated with Cm-BPH. The levels of carbonylated proteins in the extracts were quantified photometrically with 2,4-dinitrophenylhydrazine. *C*, *E. coli* KY2266 cells were transformed with an arabinose-inducible Cm-BPH gene or a Cm-BPH trap gene with mutated catalytic residues. Oxidative stress was induced by a prolonged 16 hrs stationary phase incubation, and the levels of carbonylated proteins were subsequently quantified as in *A* and *B*. The individual data points (X) and mean values +/- SD of n = 6 (*A*, *B*) or n = 3 (*C*) independent experiments are indicated.

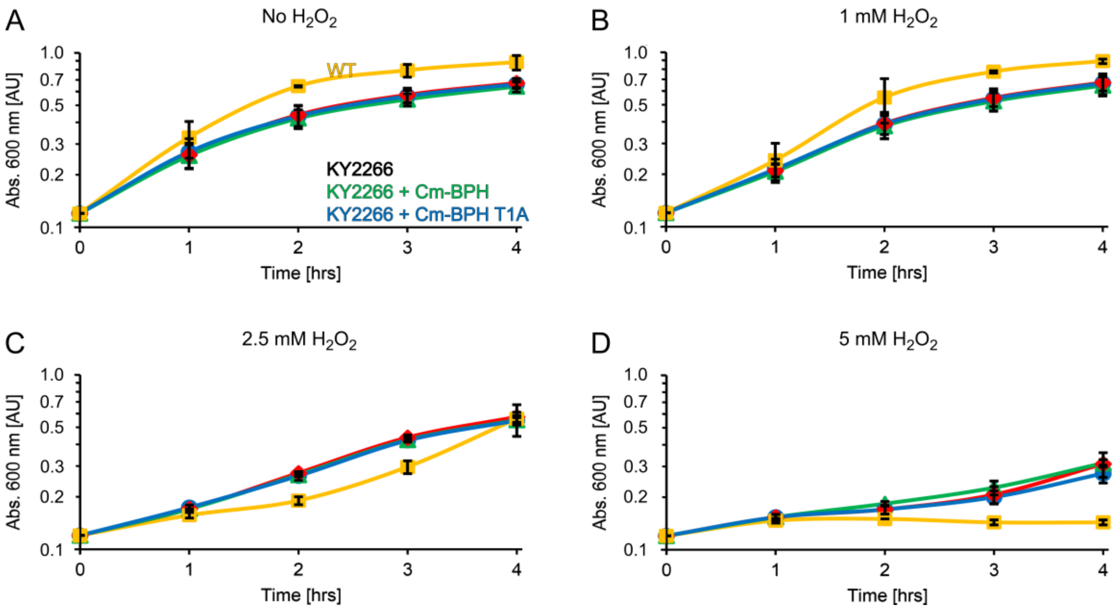

Figure S2. Effect of BPH-expression on *E. coli* growth rates in the presence of hydrogen peroxide. Growth curves for the wild type *E. coli* strain MC4100 or its mutant strain KY2266 (Δ*lon,* Δ*hslVU,* Δ*clpPX*) were recorded photometrically in the absence (*A*) or presence of 1 mM (*B*), 2.5 mM (*C*) or 5 mM (*D*) H_2O_2 and compared to KY2266 cells expressing Cm-BPH or a Cm-BPH trap mutant (see Fig. S2*C*). The values are the means +/- standard deviation of n = 3 independent experiments. The absence of cytosolic proteases leads to increased levels of heat shock proteins and stabilization of stress transcription factor σ^{32} in the mutant strain KY2266 (1), providing an explanation for its enhanced stress resistance at high peroxide concentration.

```
                                                             10        20
Alphaproteobacteria (Kiloniella litopenaei)          (M)SIVIAVKKQGKTVIGSDSKCSDI...
Betaproteobacteria (Thiobacillus sp. SCN 65-179)     (M)STVTVVRKNGRIAIAADTLTKWG...
Gammaproteobacteria (Thioploca ingrica)              (M)STVVVVKKQGIVCIAADTMTSFG...
Deltaproteobacteria (Polyangiaceae bacterium)        (M)SIAVAVRKGTTIAVAADSQENFG...
Acidobacteria (Acidobacterium Mor1)                  (M)STILVVRKGKSIAIGADTLSKHG...
Chloroflexi (Chloroflexi bacterium UTCFX4)           (M)STIIVVRKGTQACIAADSMTTWG...
Firmicutes (Clostridiales bacterium GWC2_40_7)       (M)STIVAVKKNGIAVIGADTLTKFG...
Verrucomicrobia (Pedosphaera parvula)                (M)STIVVVKKNGEVAIAADTQTTSG...
Moduliflexia (Candidatus Moduliflexus flocculans)    (M)SIAVAVQKGNDIVIAADTQDSFG...
Veturatrichia (Candidatus Vecturithrix)              (M)SIAVAVHKGQELVIAADTQDSFG...
```

Figure S3. **Alignment of BPH variants with Ser1 as putative catalytic residue, together with the phylogenetic classification of the respective organisms.** *Thiobacillus* sp. SCN 65-179 is the only known β-proteobacterial BPH variant with Ser1.

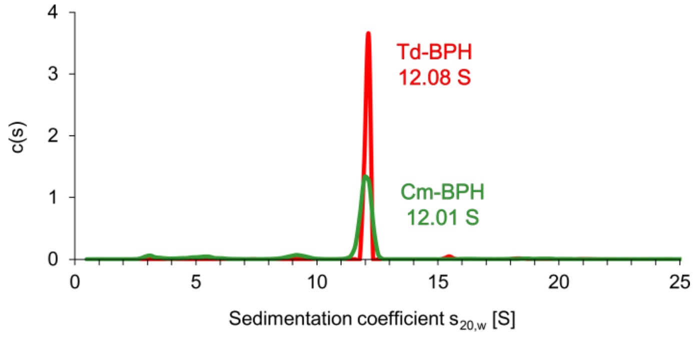

Figure S4. Sedimentation coefficient distribution of Cm-BPH (green) and Td-BPH (red) complexes, as determined by analytical ultracentrifugation.

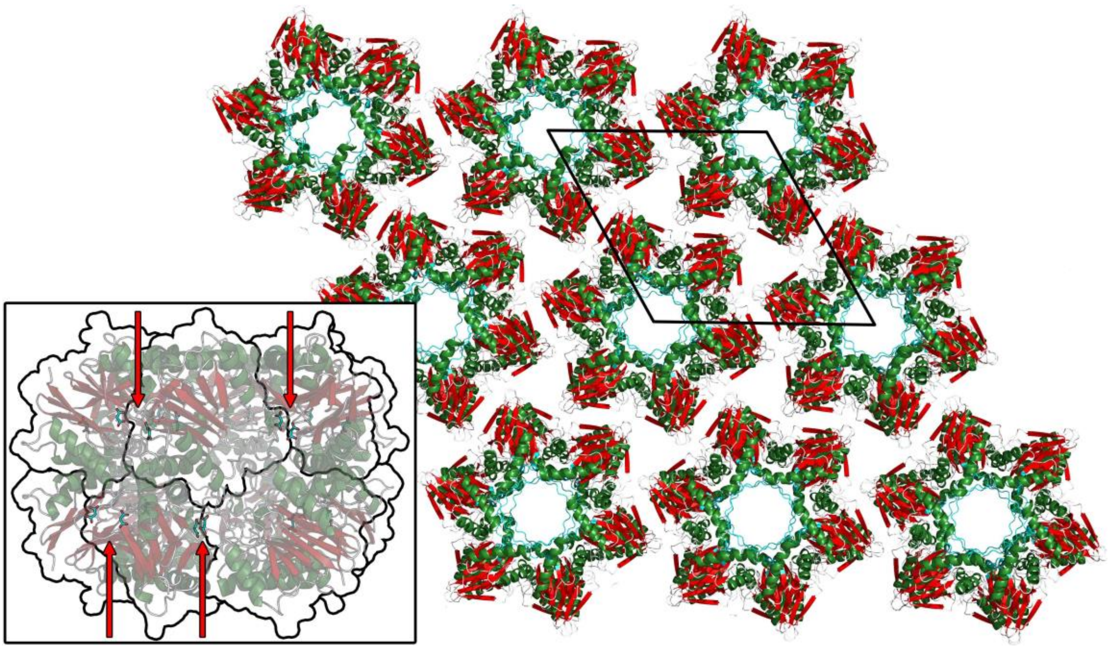

Figure S5. The dodecameric Cm-BPH assembly is stabilized by the crystal lattice and malonate from the crystallization condition. The dodecamers are constructred by crystallographic symmetry, the unit cell of the hexagonal lattice is indicated. This particular arrangement allows for an especially tight crystal packing, with a network of hydrogen bonds and bridging waters between the individual dodecamers. The inset shows the position of the malonate molecules (red arrows), which intercalate into the Cm-BPH inter-subunit clefts.

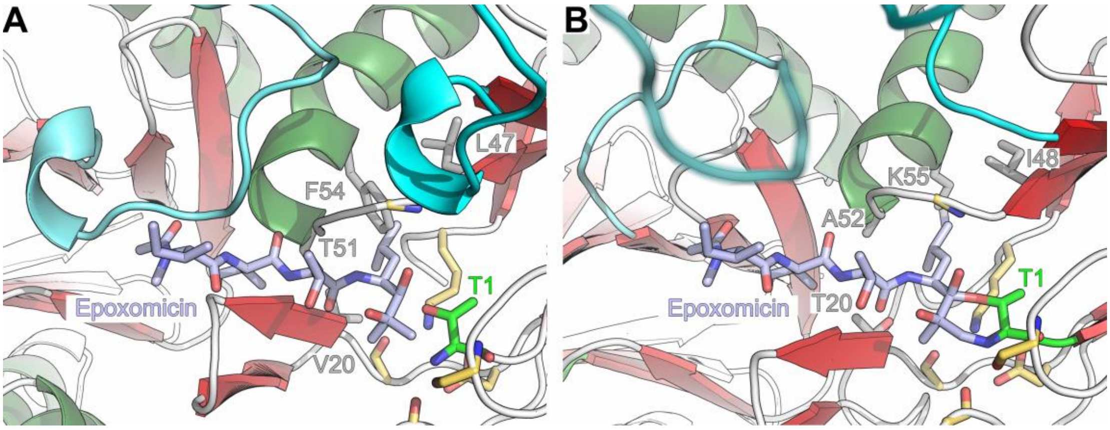

Figure S6. Comparison of pore-loop conformations. *A*, The Cm-BPH active site with an overlay of the epoxomicin molecule as it is bound in the Td-BPH active site. A clash between the pore loop and epoxomicin indicates that the pore loop conformation in the non-native Cm-BPH dodecamer is not compatible with substrate binding. *B*, Crystal structure of epoxomicin-conjugated Td-BPH, as depicted in Fig 5*C*, with a pore loop conformation permitting access to the active site.

References

1. Kanemori, M., Nishihara, K., Yanagi, H., and Yura, T. (1997) Synergistic roles of HslVU and other ATP-dependent proteases in controlling in vivo turnover of sigma32 and abnormal proteins in Escherichia coli. *J Bacteriol* **179**, 7219-7225
